# Supplementary material for: Differential mechanisms underlying responses of soil bacterial and fungal communities to nitrogen and phosphorus inputs in a subtropical forest
Source: PeerJ. 2019 Sep 6;7:e7631. doi: 10.7717/peerj.7631 (PMC6733241; doi:10.7717/peerj.7631)
Supplement: Supplemental Information 1 [file peerj-07-7631-s001.doc]

**Supporting information**

The following supporting information is available for this article:

**Table S1** Basic soil characteristics (mean ± SE, n=4) before N and P addition experiments at Jigongshan forest.

**Table S2** Environmental characteristics (plants and soil) under different treatments. Values are means of four replicate block with SE for each treatment.

**Table S3** Number of treatment-specific OTUs and of OTUs shared between two different treatments.

**Table S4** Model summary for the stepwise multiple regression of bacterial and fungal richness and diversity on environmental variables.

**Table S5** Differences in bacterial and fungal community structure between different treatments using three different statistical analyses.

**Table S6** Pearson correlations (r) between relative abundances of dominant bacterial and fungal phyla and plant and soil characteristics.

**Fig. S1** Principal composition analysis of soil bacterial (a) and fungal (b) community structure based on the OTUs profile.

**Fig. S2** Cluster analysis of the bacterial (a) and fungal (b) community according to the Bray-Curtis distance matrix

**Fig. S3** Relationships between principal component (PC1) scores of fungal community composition with PC1 scores of soil nutrients.

**Table S1** Basic soil characteristics (mean ± SE, n=4) before N and P addition experiments at Jigongshan forest.

| Soil parameters |  |
| --- | --- |
| pH (H_2_O) | 4.71 ± 0.04 |
| Total carbon (g kg^-1^) | 26.30 ± 1.77 |
| Total nitrogen (g kg^-1^) | 2.34 ± 0.13 |
| C:N ratio | 11.24 ± 2.01 |
| NH_4_^+^ (mg kg^-1^) | 7.67 ± 0.59 |
| NO_3_^-^ (mg kg^-1^) | 32.46 ± 1.80 |
| Total phosphorus (g kg^-1^) | 0.39 ± 0.20 |
| Available phosphorus (mg kg^-1^) | 18.30 ± 3.57 |

**Table S2** Environmental characteristics (plants and soil) under different treatments. Values are means of four replicate block with SE for each treatment. The bold parameters and different letters indicate significant differences between treatments (*P* < 0.05).

| Treatment | CK | N | P | NP |
| --- | --- | --- | --- | --- |
| **Fine root biomass**  **(g m^-2^)** | **178.85 ± 1.97a** | **142.57 ± 1.74d** | **159.73 ± 1.44b** | **148.59 ± 1.17c** |
| **pH** | **4.18 ± 0.08a** | **3.75 ± 0.06b** | **4.10 ± 0.15a** | **3.98 ± 0.02ab** |
| SOC (g kg^-1^) | 53.70 ± 1.20a | 53.43 ± 9.13a | 42.93 ± 13.25a | 48.10 ± 5.30a |
| TN (g kg^-1^) | 3.73 ± 0.17a | 3.15 ± 0.44a | 2.82 ± 0.72a | 2.83 ± 0.29a |
| C:N ratio | 14.53 ± 0.85a | 16.74 ± 0.73a | 14.48 ± 1.20a | 16.95 ± 0.31a |
| DOC (mg kg^-1^) | 125.00 ± 16.84a | 133.00 ± 12.38a | 99.95 ± 16.21a | 111.50 ± 10.31a |
| **NH_4_^+^ (mg kg^-1^)** | **11.59 ± 1.37b** | **23.13 ± 2.03a** | **15.37 ± 3.08b** | **18.32 ± 2.43ab** |
| NO_3_^-^ (mg kg^-1^) | 22.48 ± 3.30a | 31.33 ± 8.03a | 20.90 ± 4.00a | 23.25 ± 2.79a |
| **AP (mg kg^-1^)** | **26.28 ± 6.18b** | **40.88 ± 7.31b** | **296.75 ± 53.73a** | **285.75 ± 51.53a** |
| MBC (mg kg^-1^) | 309.75 ± 27.11a | 339.25 ± 18.37a | 325.00 ± 25.31a | 337.75 ± 15.09a |
| MBN (mg kg^-1^) | 47.93 ± 3.93a | 52.68 ± 2.01a | 52.70 ± 4.03a | 57.70 ± 3.22a |
| **Al^3+^ (mmol kg^-1^)** | **38.36 ± 3.22c** | **60.39 ± 0.40a** | **48.73 ± 0.82b** | **61.03 ± 1.42a** |
| **Ca^2+^ (mmol kg^-1^)** | **30.27 ± 2.90a** | **20.10 ± 0.53c** | **25.98 ± 0.21ab** | **22.59 ± 0.47bc** |
| Mg^2+^ (mmol kg^-1^) | 4.15 ± 1.01a | 3.48 ± 0.58a | 3.71 ± 0.11a | 3.26 ± 0.07a |
| Na^+^ (mmol kg^-1^) | 1.22 ± 0.24a | 1.32 ± 0.35a | 1.23 ± 0.07a | 1.22 ± 0.06a |

Note: TN, total nitrogen; DOC, dissolved organic carbon; AP, available phosphorus; MBC, microbial biomass carbon; MBN, microbial biomass nitrogen

**Table S3** Number of treatment-specific OTUs and of OTUs shared between two different treatments.

| Bacterial community | CK | N | P | NP |
| --- | --- | --- | --- | --- |
| CK | **25** |  |  |  |
| N | 1246 | **2** |  |  |
| P | 1397 | 1259 | **20** |  |
| NP | 1307 | 1247 | 1316 | **26** |
| Fungal community | CK | N | P | NP |
| CK | **298** |  |  |  |
| N | 1180 | **116** |  |  |
| P | 1185 | 1129 | **151** |  |
| NP | 1071 | 1060 | 1025 | **105** |

**Table S4** Model summary for the stepwise multiple regression of bacterial and fungal richness and diversity on environmental variables.

|  | Adj.r^2^ | Contribution of the individual predictor (%) | | | | |
| --- | --- | --- | --- | --- | --- | --- |
|  | Full model | pH | SOC | TN | DOC | MBC |
| **Bacterial** |  |  |  |  |  |  |
| Richness | 0.700 | 64.3 |  |  | 7.1 |  |
| Diversity | 0.866 | 50.1 |  | 14.9 | 13.4 | 9.7 |
| **Fungal** |  |  |  |  |  |  |
| Richness | 0.311 | 33.0 |  |  |  |  |
| Diversity | 0.652 | 17.0 | 19.1 |  |  | 41.4 |

**Table S5** Differences in bacterial and fungal community structure between different treatments using three different statistical analyses.

| **Bacterial community** |  | CK | N | P | NP |
| --- | --- | --- | --- | --- | --- |
| ANOSIM  r (*P* value) | CK |  |  |  |  |
|  | N | **0.875 (0.025)** |  |  |  |
|  | P | 0.031 (0.291) | 0.271 (0.137) |  |  |
|  | NP | **0.833 (0.032)** | 0.094 (0.790) | 0.186 (0.158) |  |
|  |  | CK | N | P | NP |
| ADONIS  F (*P* value) | CK |  |  |  |  |
|  | N | **4.756 (0.027)** |  |  |  |
|  | P | 1.110 (0.295) | 2.482 (0.075) |  |  |
|  | NP | **3.755 (0.029)** | 0.810 (0.713) | 1.900 (0.088) |  |
|  |  | CK | N | P | NP |
| MRPP  δ (*p* value) | CK |  |  |  |  |
|  | N | **0.318 (0.038)** |  |  |  |
|  | P | 0.356 (0.270) | 0.338 (0.089) |  |  |
|  | NP | **0.330 (0.026)** | 0.313 (0.778) | 0.350 (0.120) |  |
| **Fungal community** |  | CK | N | P | NP |
| ANOSIM  r (*P* value) | CK |  |  |  |  |
|  | N | 0.135 (0.203) |  |  |  |
|  | P | 0.156 (0.847) | 0.083 (0.573) |  |  |
|  | NP | **0.396 (0.048)** | 0.104 (0.764) | 0.099 (0.289) |  |
|  |  | CK | N | P | NP |
| ADONIS  F (*P* value) | CK |  |  |  |  |
|  | N | 1.297 (0.188) |  |  |  |
|  | P | 0.725 (0.887) | 0.804 (0.501) |  |  |
|  | NP | **2.197 (0.031)** | 0.604 (0.782) | 1.374 (0.229) |  |
|  |  | CK | N | P | NP |
| MRPP  δ (*p* value) | CK |  |  |  |  |
|  | N | 0.598 (0.187) |  |  |  |
|  | P | 0.623 (0.823) | 0.601 (0.588) |  |  |
|  | NP | **0.546 (0.034)** | 0.524 (0.840) | 0.548 (0.224) |  |

**Table S6** Pearson correlations (r) between relative abundances of dominant bacterial and fungal phyla and plant and soil characteristics.

| r | Fine root biomass | pH | SOC | TN | C:N ratio | DOC | NH_4_^+^ | NO_3_^-^ | AP | MBC | MBN | Al^3+^ | Ca^2+^ | Mg^2+^ | Na^+^ |
| --- | --- | --- | --- | --- | --- | --- | --- | --- | --- | --- | --- | --- | --- | --- | --- |
| **Bacterial phylum** |  |  |  |  |  |  |  |  |  |  |  |  |  |  |  |
| *Proteobacteria* | -0.014 | -0.309 | 0.246 | 0.097 | **0.519*** | 0.037 | 0.218 | 0.243 | -0.077 | 0.158 | 0.194 | 0.050 | 0.188 | -0.300 | 0.101 |
| *Acidobacteria* | -0.199 | 0.098 | -0.003 | -0.083 | 0.113 | -0.091 | -0.114 | -0.245 | 0.147 | -0.172 | 0.043 | 0.134 | -0.302 | 0.272 | -0.362 |
| *Actinobacteria* | -0.010 | -0.193 | 0.131 | 0.334 | -0.301 | **0.583*** | 0.022 | 0.164 | -0.174 | 0.350 | 0.012 | 0.083 | -0.090 | 0.039 | 0.140 |
| *Planctomycetes* | 0.088 | 0.419 | -0.390 | -0.410 | -0.206 | **-0.553*** | 0.063 | -0.041 | 0.387 | -0.141 | 0.042 | -0.222 | 0.186 | -0.341 | 0.243 |
| *Chloroflexi* | **0.583*** | **0.682**** | -0.328 | -0.153 | **-0.666**** | **-0.502*** | -0.457 | -0.476 | 0.057 | **-0.517*** | -0.459 | **-0.675**** | 0.376 | 0.435 | -0.165 |
| *Firmicutes* | **-0.543*** | -0.415 | 0.034 | -0.172 | 0.305 | 0.043 | 0.380 | 0.247 | 0.085 | 0.254 | 0.181 | **0.585*** | -0.352 | -0.377 | 0.377 |
| *Bacteroidetes* | 0.199 | -0.021 | 0.135 | 0.133 | 0.263 | 0.001 | 0.177 | 0.375 | 0.060 | 0.111 | 0.149 | -0.134 | 0.285 | -0.376 | 0.095 |
| *Gemmatimonadetes* | **0.541*** | **0.735*** | -0.487 | -0.424 | -0.449 | **-0.741**** | -0.496 | -0.468 | -0.048 | **-0.723**** | **-0.513*** | **-0.517*** | 0.412 | 0.376 | -0.070 |
| *Verrucomicrobia* | 0.173 | 0.254 | -0.336 | -0.238 | **-0.532*** | -0.290 | -0.073 | -0.127 | 0.091 | -0.190 | -0.244 | -0.154 | 0.137 | -0.065 | -0.157 |
| *Armatimonadetes* | -0.287 | -0.131 | -0.363 | **-0.509*** | 0.139 | **-0.516*** | 0.217 | 0.023 | 0.179 | -0.203 | 0.035 | 0.164 | -0.238 | -0.223 | 0.262 |
| *Elusimicrobia* | 0.423 | 0.349 | -0.114 | 0.012 | -0.345 | -0.230 | -0.484 | -0.433 | **-0.535*** | **-0.602*** | **-0.591*** | -0.470 | 0.167 | **0.531*** | -0.326 |
| *Cyanobacteria* | -0.262 | -0.098 | 0.004 | -0.242 | 0.479 | -0.165 | 0.161 | 0.037 | 0.060 | 0.101 | 0.328 | 0.274 | -0.039 | -0.461 | 0.334 |
| *Dependentiae* | -0.030 | 0.384 | **-0.564*** | **-0.682**** | -0.077 | **-0.697**** | 0.207 | 0.147 | 0.016 | -0.469 | -0.208 | -0.075 | 0.112 | -0.324 | 0.001 |
| *Saccharibacteria* | **-0.651*** | **-0.715*** | 0.341 | 0.149 | **0.537*** | 0.380 | **0.717**** | **0.574*** | -0.129 | 0.473 | 0.362 | **0.609*** | **-0.534*** | -0.347 | 0.335 |
| *Omnitrophica* | **0.604*** | 0.132 | 0.369 | 0.451 | 0.054 | -0.004 | -0.285 | -0.147 | 0.073 | 0.003 | 0.004 | **-0.573*** | **0.568*** | 0.129 | -0.075 |
| **Fungal phylum** |  |  |  |  |  |  |  |  |  |  |  |  |  |  |  |
| *Ascomycota* | 0.267 | **0.593*** | **-0.697**** | **-0.641**** | -0.368 | **-0.603*** | -0.144 | -0.076 | -0.227 | **-0.697**** | -0.465 | -0.314 | 0.238 | -0.156 | -0.243 |
| *Basidiomycota* | 0.479 | 0.479 | -0.150 | 0.073 | -0.482 | -0.011 | **-0.626**** | **-0.566*** | -0.309 | -0.452 | -0.487 | -0.400 | 0.150 | **0.562*** | -0.330 |
| *Zygomycota* | 0.138 | 0.013 | 0.129 | 0.098 | 0.155 | 0.053 | -0.175 | -0.095 | 0.100 | 0.117 | 0.021 | -0.111 | 0.308 | -0.212 | 0.037 |
| *Rozellomycota* | 0.319 | 0.194 | -0.163 | -0.148 | -0.065 | -0.021 | -0.353 | -0.179 | -0.364 | -0.209 | -0.038 | -0.052 | 0.456 | 0.118 | -0.011 |

*: 0.01 < P ≤ 0.05; **: 0.001 < P ≤ 0.01; *** P ≤ 0.001


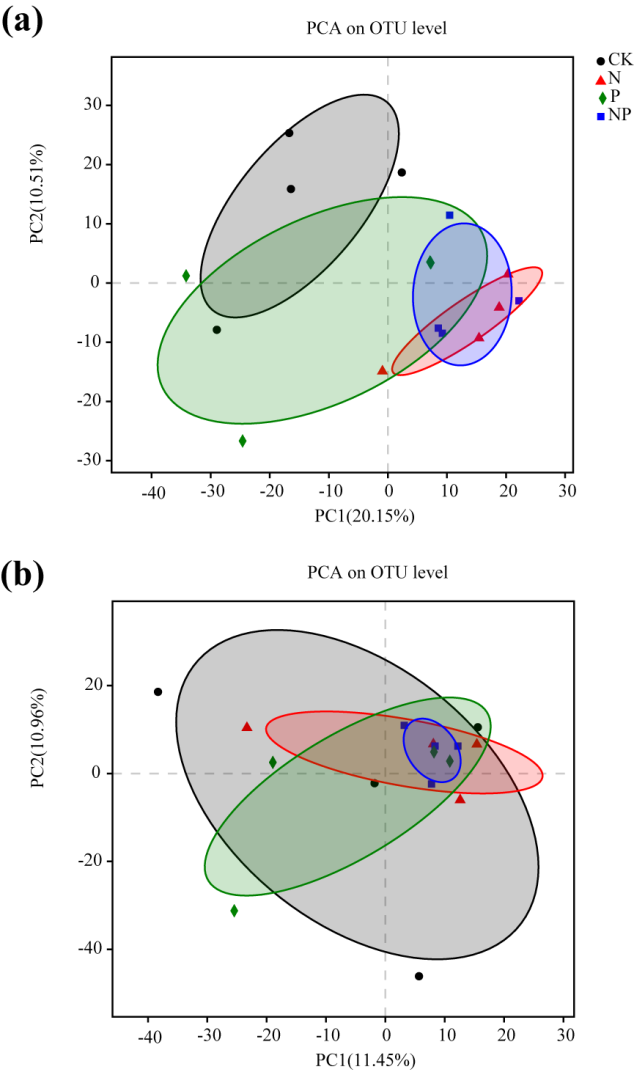


**Fig. S1** Principal composition analysis of soil bacterial (a) and fungal (b) community structure based on the OTUs profile.

**
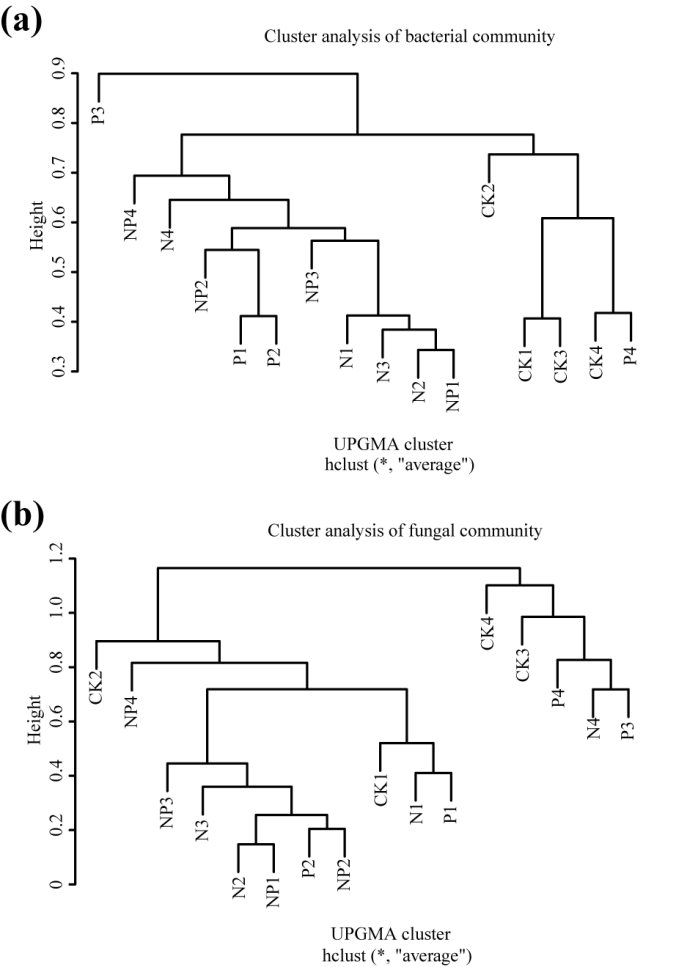
**

**Fig. S2** Cluster analysis of the bacterial (a) and fungal (b) community according to the Bray-Curtis distance matrix

**Fig. S3** Relationships between principal component (PC1) scores of fungal community composition with PC1 scores of soil nutrients.
